# Clinical significance of Janus Kinase inhibitor selectivity

**DOI:** 10.1093/rheumatology/key339

**Published:** 2018-12-01

**Authors:** Ernest H Choy

**Affiliations:** CREATE Centre, Section of Rheumatology, Division of Infection and Immunity, Cardiff University School of Medicine, Cardiff, UK

**Keywords:** rheumatoid arthritis, Janus Kinase, treatment, targeted synthetic DMARDs, DMARDs

## Abstract

Cytokines are key drivers of inflammation in RA, and anti-cytokine therapy has improved the outcome of RA. Janus Kinases (JAK) are intracellular tyrosine kinases linked to intracellular domains of many cytokine receptors. There are four JAK isoforms: JAK1, JAK2, JAK3 and TYK2. Different cytokine receptor families utilize specific JAK isoforms for signal transduction. Phosphorylation of JAK when cytokine binds to its cognate receptor leads to phosphorylation of other intracellular molecules that eventually leads to gene transcription. Oral JAK inhibitors (JAKi) have been developed as anti-cytokine therapy in RA. Two JAKi, tofacitinib and baricitinib, have been approved recently for the treatment of RA, and many JAKi are currently in development. JAKi inhibit JAK isoforms with different selectivity. This review discusses the efficacy and safety of JAKi in RA, in particular the potential clinical significance of JAKi selectivity.

**Rheumatology key messages**

- Janus Kinase inhibitor selectively is relative and not absolute.
- Current approved Janus Kinase inhibitors and those in development significantly inhibit Janus Kinase 1 isoform.
- Janus Kinase 1 is an effective target in RA, although zoster reactivation is a class effect.

## Background

During the 1990s, when mAbs were trialled in patients with RA, many rheumatologists questioned whether i.v. or s.c. injections could ever be a realistic treatment. At the time, researchers argued that the mAbs were the ideal tools to identify specific therapeutic targets for which oral inhibitors can be developed. This vision was realized when in 2009 the Food and Drug Administration approved the first Janus Kinase inhibitor (JAKi), tofacitinib, for the treatment of RA. In 2017, tofacitinib and another JAKi, baricitinib, received approval in Europe. Janus Kinases (JAKs) are intracellular molecules involved in signal transduction of type I and II cytokine receptors [1] including IL-6 receptor, a proven therapeutic target in RA [2]. JAKi are classified by EULAR as targeted synthetic DMARDs. There are four JAK isoforms: JAK1, JAK2, JAK3 and TYK2, which act in pairs to phosphorylate other intracellular proteins. Other JAKi are being developed for the treatment of RA. Current JAKi that are approved and in development for RA inhibit JAK isoforms with different selectivity. Is JAK selectivity clinically meaningful? This review will discuss the efficacy and safety of JAKi in RA as well as the potential clinical significance of JAK isoform selectivity.

### Cytokine signalling and biology of the JAK/STAT pathway

Cytokines can be classified by the structure of their receptors. Type I cytokine receptors have certain conserved motifs in their extracellular amino acid domain. These include common γ chain (such as IL-2), gp130 family (such as IL-6), p40 subunit (IL-12 and IL-23) and common β chain cytokine receptors (haemopoietic cytokines such as GM-CSF). Type II cytokine receptors are members of the IL-10 and IFN families. Both Type I and II cytokine receptor families utilize the JAK for signalling transduction. Among these cytokines and cytokine receptors, IL-6 and IL-6 receptor are established therapeutic targets in RA. The role of other JAK signalling cytokines in RA is less well established. JAKs also have essential homeostatic roles, being responsible for signalling of some hormones including prolactin and growth hormone.

**Fig. 1:**
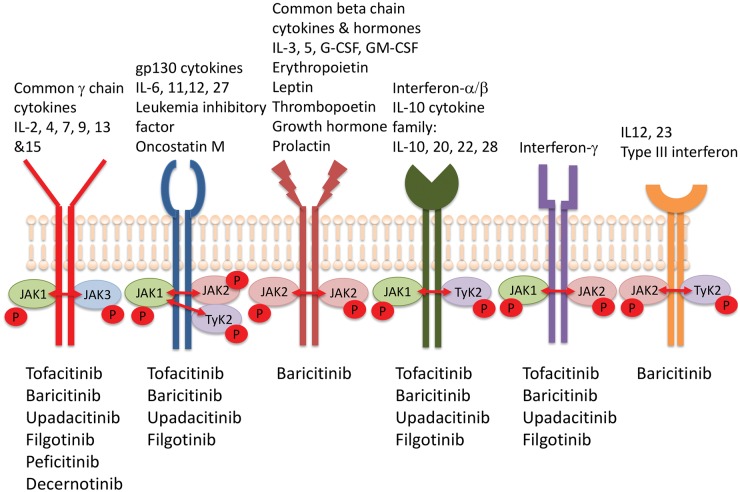
Cytokine signalling via JAK isoforms and their inhibitors JAK: Janus Kinase; p: phosphate.

JAKs are associated with the membrane proximal regions of cytokine receptors [3]. Conformational changes in cytokine receptors induced by ligand binding lead to phosphorylation of JAKs through the reciprocal interaction of two juxtapositioned JAKs (Fig. 1). Hence, JAK activation requires two JAK isoforms either as homodimers or heterodimers to auto-phosphosphorylate, which allows recruitment and phosphorylation of various signalling molecules including members of the signal transducer and activator of transcription (STAT) family of DNA binding proteins [4]. Phosphorylation of STATs promotes their translocation to the nucleus and gene transcription.

Since type I and II cytokine receptor families include a large number of cytokines, growth factors and hormones, JAKs are critical to immune function and homeostasis. Consequently, JAKs are highly conserved and JAK isoforms are non-redundant. JAK isoform knock-out animals have severe clinical phenotypes: JAK1-deficient mice die perinatally [5] while JAK2 knockout animals are embryonic lethal due to defective erythropoiesis [6]. JAK3-deficient mice suffer from severe immunodeficiency syndrome and reduced survival [7]. In humans, mutations in JAK3 cause severe combined immune deficiency syndrome [8, 9]. TYK2-deficient mice are viable but are susceptible to viral infection due to reduced IFN response [10].

### Therapeutic principle of JAK inhibition

Evidence from animals and patients with JAK isoform deficiency showed that complete blockade of JAK isoforms is undesirable as it will lead to severe immunodeficiency and abnormal homeostasis. Therefore, the principle of JAK inhibition differs from cytokine inhibition using biologics in that the objective is not to specifically block the JAK pathway completely but to reversibly reduce the activity of one or more JAK isoforms, akin to turning down a thermostat. One potential clinical advantage of such a mode of action is that inhibition can be rapidly reversed with ‘fast-on’ and ‘fast-off’ effects.

### JAKi

Two JAKi, tofacitinib and baricitinib, in combination with MTX or as monotherapy, have been approved for the treatment of RA. Both tofacitinib and baricitinib have been examined in large phase III and IV randomized control trials (RCT) in a range of RA patients from conventional synthetic DMARD (csDMARD)-naïve early patients, csDMARD inadequate responders to biologic inadequate responders. The ACR responses in these studies are summarized in Table 1. Tofacitinib is selective for JAK1 for JAK3, while baricitinib is selective for JAK1 and JAK2. In addition, four JAKi are currently in development: the JAK1 selective upadacitinib and filgotinib, the JAK1 and JAK2 inhibitor peficitinib, and the JAK3 selective inhibitor decernotinib.

**Table 1:** ACR responses at primary endpoints in tofacitinib and baricitinib RCTs

	Controls			JAKi			Active comparator		
	ACR20	ACR50	ACR70	ACR20	ACR50	ACR70	ACR20	ACR50	ACR70
MTX inadequate responders									
Oral-STANDARD [11] Tofacitinib (5 mg bid)	26	7	2	61	34	12	56	24	9
Oral-STANDARD [11] Tofacitinib (10 mg bid)				59	28	15			
RA-BEAM [12] Barcitinib (4 mg qd)	40	17	5	70	45	19	61	35	13
cDMARD inadequate responders									
Oral-SYNC [13] Tofacitinib (5 mg bid)	27	10	2	56	27	8			
Oral-SYNC [13] Tofacitinib (10 mg bid)				65	34	14			
RA-BUILD [14] Barcitinib (2 mg qd)	39	13	3	66	34	18			
RA-BUILD [14] Barcitinib (4 mg qd)				62	33	18			
bDMARD inadequate responders									
Oral-STEP [15] Tofacitinib (5 mg bid)	24	8	2	42	27	14			
Oral-STEP [15] Tofacitinib (10 mg bid)				48	28	11			
RA-BEACON [16] Barcitinib (2 mg qd)	27	8	2	49	20	13			
RA-BEACON [16] Barcitinib (4 mg qd)				55	28	11			
MTX naïve									
Oral-START [17] Tofacitinib (5 mg bid)	51 (MTX as control)	27 (MTX as control)	12 (MTX as control)	71	47	26			
Oral-START [17] Tofacitinib (10 mg bid)				76	56	38			
RA-BEGIN [18] Barcitinib (4 mg qd)+MTX	62 (MTX as control)	43 (MTX as control)	21 (MTX as control)	78	63	40			
Monotherapy									
Oral-SOLO [19] Tofacitinib (5 mg bid)	27	13	6	60	31	15			
Oral-SOLO [19] Tofacitinib (10 mg bid)				66	37	20			
RA-BEGIN [18] Barcitinib (4 mg qd)	62 (MTX as control)	43 (MTX as control)	21 (MTX as control)	77	60	42			

Numbers are percentages. JAKi: Janus Kinase inhibitor; RCT: randomized control trial; bDMARD: biological DMARD.

In MTX inadequate responders, tofacitinib [5 and 10 mg bis in die (bid)] in Oral-STANDARD [11] and baricitinib [4 mg once daily (od)] in RA-BEAM [12] added to MTX showed superior ACR responses when compared with placebo. Tofacitinib showed a similar response to adalimumab while baricitinib in RA-BEAM achieved statistically significant higher ACR20 responses than adalimumab (70 *vs* 61%). However, the difference was small (<10%) and the sample size was larger in RA-BEAM than in Oral-STANDARD. Similar to MTX inadequate responders, in csDMARD inadequate responders, adding tofacitinib in Oral-SYNC [13] and baricitinib in RA-BUILD [14] achieved higher ACR responses than placebo. In biologic inadequate responders, tofacitinib (5 and 10 mg bid) in Oral-Step [15] and baricitinib (2 and 4 mg od) in RA-BEACON [16] in combination with MTX achieved higher ACR responses than placebo.

### Radiographic damage

In ORAL-SCAN [20], radiographic damage was statistically significantly less in patients treated with tofacitinib 10 mg when compared with placebo-treated patients. Tofacitinib 5 mg-treated patients had less radiographic damage than placebo-treated patients but this did not achieve statistical significance. Baricitinib has also been shown to reduce radiographic damage in RA-BUILD [14], RA BEAM [12] and RA-BEGIN [18]. In RA-BUILD, baricitinib, both 2 and 4 mg in combination with MTX statistically significantly reduced radiographic progression when compared with placebo. In RA-BEGIN, baricitinib 4 mg monotherapy-treated patients had less radiographic progression than placebo but the difference was not statistically significant.

### Monotherapy vs combination therapy with MTX

Since JAKi are not biological DMARDs, they do not incite an anti-drug antibody response so theoretically concomitant treatment with MTX should be unnecessary. Tofacitinib monotherapy was assessed in Oral-SOLO [19] and Oral-START [17], while baricitinib monotherapy was assessed in RA-BEGIN [18]. Tofacitinib (5 and 10 mg) and baricitinib 4 mg monotherapy were superior to MTX. Barcitinib monotherapy produced a similar therapeutic response to 4 mg plus MTX. However, the sample size of the study was not powered to compare difference between monotherapy *vs* combination therapy. Indeed, the sample size of the monotherapy was smaller (*N* = 159) than the MTX plus baricitinib group (*N* = 215). Furthermore, both Oral-START and RA-BEGIN were trials of patients with early RA while in routine clinical practice, JAKi are used in patients with established disease. These studies showed than JAKi monotherapy is effective, but it is unclear whether monotherapy is as effective as combination therapy. For tofacitinib, this was assessed in ORAL-STRATEGY [21], a 1-year, double-blind, head-to-head, non-inferiority, RCT comparing tofacitinib (5 mg bid) monotherapy, tofacitinib (5 mg bid) plus MTX, and subcutaneous adalimumab (40 mg fortnightly) plus MTX in MTX inadequate responder patients. The primary endpoint was ACR50 response at month 6. This was met by 38, 46 and 44% of patients in tofacitinib monotherapy, tofacitinib plus MTX and adalimumab plus MTX, respectively. Tofacitinib plus MTX was non-inferior to adalimumab plus MTX but non-inferiority was not demonstrated in the tofacitinib monotherapy group, suggesting that in patients who can tolerate MTX, combining tofacitinib with MTX is better than switching to monotherapy.

### JAKi in development

Phase II RCT data of upadacitinib [22, 23], filgotinib [24, 25], peficitinib [26, 27] and decernotinib [28, 29] are summarized in Table 2. Overall, these JAKi demonstrated superior ACR responses than placebo-treated group. Recently, phase III trials of upadacitinib in csDMARD inadequate responders (SELECT Next) [30] and biologic inadequate responder (SELECT Beyond) [31] patients have been published that confirmed the efficacy of updacitinib (15 and 30 mg od).

**Table 2:** Results of phase II RCT of JAKi in development

		Controls			JAKi		
		ACR20	ACR50	ACR70	ACR20	ACR50	ACR70
JAK1 selective							
FilgotinibDARWIN 1 [24] (+ MTX)	50 mg qd	44	15	8	56	33	16
	100 mg qd				64	38	21
	200 mg qd				69	43	24
FilgotinibDARWIN 2 [25] (monotherapy)	50 mg qd	29	11	4	67	35	8
	100 mg qd				67	36	19
	200 mg qd				72	43	13
UpadacitinibBALANCE 2 [23] (MTX inadequate responders)	6 mg bid	46	18	6	68	46	28
	12 mg bid				80	50	16
	18 mg bid				64	40	26
UpadacitinibBALANCE 1 [22] (TNF inadequate responders)	6 mg bid				58	36	26
	12 mg bid				71	42	22
	18 mg bid				67	38	22
Moderate JAK3 selective							
Dercernotinib [28]	100 mg qd	18	7	3	47	23	10
	150 mg qd				67	39	11
	200 mg qd				57	35	10
	100 mg bid				68	39	22
Dercernotinibmonotherapy [27]	25 mg bid	29	7	2	39	17	7
	50 mg bid				61	32	12
	100 mg bid				65	38	18
	150 mg bid				66	49	22
Peficitinib [27]	25 mg qd	44	26	11	44	18	9
	50 mg qd				62	33	15
	100 mg qd				46	33	17
	150 mg qd				57	37	19
Peficitinib [26]	25 mg qd	29	10	8	22	15	7
	50 mg qd				37	25	16
	100 mg qd				48	28	19
	150 mg qd				56	28	11

Numbers are percentages. bid: bis in die (twice per day); JAK: Janus Kinase; JAKi: Janus Kinase inhibitor; RCT: randomized control trial; qd: quaque die (once per day).

### Safety profile of JAKi

Safety data on JAKi are mostly based on RCT and extension studies. Limited real-world data are available for tofacitinib [32]. Caution should be exercised when comparing the incidence rate (IR) of adverse events with biologic agents, in which IR from real-world data [33] are available. In pooled analyses, the IR of serious adverse events was 9.4/100 patient years (95% CI 9.0–0.9) for tofacitinib [34]. For baricitinib, the IR of severe adverse events has not been reported but the IR of specific adverse events are available. The key adverse event data on tofacitinib and baricitinib are summarized in Table 3.

**Table 3:** Safety of tofacitinib and baricitinib

	Tofacitinib (JAK, JAK3)	Baricitinib (JAK1, JAK2)	Peficitinib (JAK1, JAK2)	Fligotinib (JAK1)	Upadacitinib (JAK1)	Decernotinib (JAK3)
Serious infection	2.7 (2.5–3.9)	2.9 (2.5–3.4)				
Herpes zoster	3–4					
Malignancies	0.9(0.8–1)	0.8 (0.6–1.0)				
Lymphoma	0.1 (0.1–0.2)	0.09 (0.03–0.19)				
Non-melanoma skin cancer	0.6 (0.5–0.7)	0.4 (0.2–0.5)				
Gastrointestinal perforation	0.11 (0.07–0.17)	0.05 (0.01–0.13)				
DVT/PE	NR	0.5 (0.3–0.7)				
Changes in laboratory tests (mean + s.d.)						
Haemoglobin (g/dl)	+0.47 + 0.05 (5 mg), +0.28 + 0.05 (10 mg)	−0.17	Decrease	Increase	Decreases at higher doses	Increase
Neutrophil (×10^3^/mm^3^)	−1.09 + 0.1 (5 mg), −1.49 + 0.1 (10 mg)	−1.08 +0.07	Decrease	Decrease	Decrease	Decrease in high dose
Lymphocyte count (×10^3^/mm^3^)	−0.24 + 0.03 (5 mg), −0.36 + 0.03 (10 mg)	−0.01 (2 mg) −0.05 (4 mg)	Decrease	No change; some patients developed lymphopaenia	Decrease	Decrease
Platelets	−30%	Increase	Decrease	Decrease	NR	NR
Liver transaminase	Increase	Increase	Increase in high dose group	Increase	Increase	Increase
Cholesterol	Increase	Increase	Increase	Increase	Increase	Increase
Creatinine	Increase	Increase	Increase	Increase	Increase	Increase
Creatinine phosphokinase	Increase	Increase	Increase	NR	Increase	Increase

Safety data are incidence rate per 100 patient years with 95% CI. Laboratory results are mean ± s.d. unless stated otherwise. JAK: Janus Kinase; NR: not reported; DVT/PE: deep vein thrombosis and pulmonary embolus.

### Infections

The IR of serious infections was 2.7/100 patient years (95% CI 2.5–3.9) [29] and 2.9/100 patient years (95% CI 2.5–3.4) [35] for tofacitinib and baricitinib, respectively. Both tofacitinib and baricitinib were associated with increased incidence of reactivation of herpes zoster (3–4/100 patient years). This was higher than placebo and exceeded those expected with biologic agents. Risk was highest in Japan and Korea [36]. Concomitant glucocorticoid was an additional risk factor. Reactivation of herpes zoster appears to be a class effect and may be due to inhibition of IFN and IL-15, which are key anti-viral cytokines that signal through JAK1, JAK2 and JAK3. Response to zoster vaccine prior to JAKi treatment has been evaluated in an RCT [37]. Zoster vaccine was given to 112 patients with active RA taking MTX for 2 weeks before receiving either placebo or tofacitinib for 12 weeks, after which placebo-treated patients received tofacitinib in an open-label extension study. Both humoral and cellular immune responses to the zoster vaccine were similar in placebo- and tofacitnib-treated patients. In the open-label extension phase, five patients developed reactivation of zoster. All these patients had suboptimal vaccine immune response to the zoster vaccine.

### Malignancies

IR of malignancies excluding non-melanoma skin cancer for tofacitinib [29] and baricitinib [30] were 0.9/100 patient years (95% CI 0.8–1) and 0.8/100 patient years (95% CI 0.6–1.0), respectively. IR for lymphoma was 0.1/100 patient years (95% CI 0.1–0.2) for tofacitinib and 0.09/100 patient years (95% CI 0.03–0.19) for baricitinib. Non-melanoma skin cancer occurred in 0.6/100 patient years (95% CI 0.5–0.7) for tofacitinib and 0.4/100 patient years (95% CI 0.2–0.5) for baricitinib. However, long-term real-world data will be needed to assess accurately the risk of malignancies.

### Gastrointestinal perforation

Gastrointestinal perforation is associated with IL-6 inhibition. IL-6 signals via JAK1, JAK2 and TYK2. Therefore, inhibiting IL-6 signalling by JAKi may be associated with gastrointestinal perforation. IR of gastrointestinal perforation was 0.11/100 patient years (95% CI 0.07–0.17) for tofacitinib and 0.05/100 patient years (95% CI 0.01–0.13) for baricitinib. These were numerically lower than that was observed with tocilizumab reported in German biologic registry, which was 0.27/100 patient years [38]. Indeed, a real-world study also suggested the same. Lower gastrointestinal tract perforation was slightly less frequent in tofacitinib-treated than in tocilizumab-treated patients, although the risk was higher than in patients treated by TNF inhibitors [39].

### Deep vein thrombosis and pulmonary embolus

Five cases of deep vein thrombosis and pulmonary embolus (DVT/PE) were observed in baricitinib- (IR 1.2/100 patient years) but none in the placebo-treated patients during RCTs [40]. The overall IR of DVT/PE was 0.5/100 patient years (95% CI 0.3–0.7). There was no association between platelet count and the occurrence of DVT/PE. The IR of DVT and PE associated with tofacitinib has not been reported. However, as these adverse events are uncommon, data from registries will be needed to evaluate the association between DVT/PE and JAKi.

### Laboratory abnormalities

#### Haemoglobin

One of the extra-articular features of active RA is anaemia of chronic diseases, which is mediated by hepcidin as part of the acute-phase response [2]. Hence, effective suppression of inflammation should increase haemoglobin (Hb), which has been seen with biologic treatment [41, 42]. However, haemopoietic cytokines including erythropoietin signal via JAK2 [43]. A consequence of JAK2 inhibition is reduced erythropoiesis. This is reflected by Hb changes associated with baricitinib [44], in which a statistically significant greater reduction (*P* = 0.02) in Hb occurred in patients treated with baricitinib (−0.17 ± 0.02) when compared with placebo-treated patients (−0.12 ± 0.02). Anaemia occurred in 29% of baricitinib-treated *vs* 26% of placebo-treatment patients. In contrast, a small increase in Hb was observed in a pooled analysis of tofacitinib, which has less inhibitory effect on JAK2: 0.47 g/dl and 0.28 g/dl with 5 and 10 mg, respectively [45]. The likely reason for a smaller increase in Hb with tofacitinib 10 mg is dose-associated inhibition of JAK2, i.e. at low dose (5 mg) tofacitinib is selective for JAK1 and JAK3 but at 10 mg, this selectivity is diminished and JAK2 is inhibited. Compared with MTX, both doses of tofacitinib were associated with a slightly higher incidence of anaemia, although in total <1% of patients experienced major decrease in Hb as defined by decrease from baseline of ⩾3 g/dl or an absolute haemoglobin level of ⩽7 g/dl. Nevertheless, the Summary of Product Characteristics recommends that tofacitinib [46] and baricitinib [47] should not be used in patients who are anaemic (Hb <8g/dl) and treatment should be interrupted when Hb drops below 8 g/dl.

#### Neutrophil

Decrease in neutrophil count with occasional cases of neutropaenia (Table 3) has been observed with all JAKi. The Summary of Product Characteristics recommends monitoring of neutrophil count in patients taking JAKi [46, 47].

#### Lymphocyte

Lymphopaenia can occur in patients treated with JAKi. For tofacitinib, lymphopaenia <500 cells/ml occurred in 8.3/100 patient years (95% CI 3.0–18.1) [39]. Multivariate analysis suggested that lymphocyte count <500 cells/ml was associated with increased risk of serious infection [29]. Lymphopaenia was uncommon (<1% of patients) but has also been observed after treatment with baricitinib [48]. The presence of lymphopaenia was associated with a slightly higher overall infection rate, but not for serious infections; however, the lack of association with serious infection may be due to insufficient event and patient number. The Summary of Product Characteristics recommends interrupting JAKi treatment when lymphocyte count is <500 cells/ml [46, 47].

#### Platelet

Thrombocytosis is a feature of active disease in RA. Suppression of inflammation should reduce platelet number. Surprisingly, treatment by baricitinib was associated with thrombocytosis [41]. Increases in platelet counts above 600×10^9^ cells/l occurred in 2.0% of patients compared with 1.1% of placebo-treated patients [47]. There are no published data on change in platelet count after treatment with tofacitinib.

### Biochemistry

#### Lipids

Increase in both high-density lipoprotein and low-density lipoprotein cholesterol occurred after treatment with both tofacitinib [49] and baricitinib [50], although there was no change in high-density lipoprotein/low-density lipoprotein ratio. IR of major adverse cardiovascular events was 0.58/100 patient years (95% CI 0.39–0.88) for tofacitinib [51] and 0.5/100 patient years (95% CI 0.4–0.7] for baricitinib [40]. A mechanistic study examined the effect of tofacitinib on cholesterol and lipoprotein transport showed that high-density lipoprotein, low-density lipoprotein and total cholesterol levels were lower in

 RA patients than in healthy volunteers, while the cholesterol ester fractional catabolic rate was higher in RA patients [52], suggesting cholesterol catabolism is increased. Treatment with tofacitinib reduced inflammation and restored cholesterol catabolism.

#### Liver transaminases

Increases in liver transaminases (>3× upper limit of normal) occurred in up to 2% of patients receiving tofacitinib [53] and 1.4% of patients treated with baricitinib [47]. Most cases were transient and asymptomatic. Liver function tests should be monitored in patient taking JAKi and when liver transaminases increase significantly, treatment should be temporarily discontinued.

#### Increase in creatinine levels

A small increase in serum creatinine by 2–4 µmol/l was observed in RCTs when compared with placebo for tofacitinib [54] and baricitinib [55]. This plateaued after 3 months and was not associated with significant renal side effects.

#### Increase in creatine phosphokinase

Increase in creatine phosphokinase (>5× the upper limit of normal) occurred in up to 1% of patients treated with either tofacitinib [46] or baricitinib [47]. In most cases, creatine phosphokinase elevation was transient, asymptomatic and did not require treatment discontinuation.

#### Safety of JAKi in development

Exposure is too low to assess major clinical safety such as serious infection. For laboratory measures, changes in biochemistry measures have been observed with all JAKi, suggesting that these are class effects (Table 3).

For haematology measures, decreases in neutrophil and lymphocyte count, including neutropaenia and lymphopaenia, have been observed with all JAKi, suggesting that these are also class effects. However, JAKi have different effects on Hb and platelet counts. Filgotinib and decernotinib increased Hb but peficitinib and upadacitinib, especially at high doses, decreased Hb. Therefore, filgotinib and decernotinib are similar to tofacitinib, while peficitinib and upadacitinib are more akin to baricitinib. The most likely explanation for these differences is inhibition of JAK2, which is expected from the *in vitro* profile of baricitinib and peficitinib. Another potential differentiating feature among JAKi is the effect on platelet count: both filgotinib and peficitinib decrease platelet numbers while baricitinib increases platelet number. The effect of upadacitinib and decernotinib on platelet number was not reported.

#### JAK isoform selectivity

Structurally, JAKs are composed of seven homologous regions, JH1–7. JH5–7 are critical for the association of the JAKs with their cognate receptors while JH1 kinase is the active catalytic domain and the main target for JAKi. Because the JH1 domains of the JAK isoforms exhibit a high degree of homology, JAKi are selective but not specific. JAKi selectivity is assessed by *in vitro* assay of cytokine release and/or pSTAT activation. It is important to note that results from these assays depend on the assay substrates/cell lines and cytokines measured. Furthermore, JAK1, JAK2 and TYK2 are ubiquitously expressed, but JAK3 is predominantly expressed by haematopoietic cells and is highly regulated with cell development and activation.

#### JAK isoform selectivity is dose dependent

Table 4 shows the concentration needed to inhibit 50% of activation (IC50) of different JAKi. A low IC50 value implies higher potency. JAK isoform selectivity is determined by ratio and difference between IC50s for different JAK isoforms. Table 4 shows not only the IC50 but also the ratio of IC50s. One may consider the ratio as the **‘**JAK selectivity dose window’. Conventional pharmacology will select compounds with high potency; however, in the case of JAKi, potency affects the JAK selectivity dose window. Highly potent compounds will have narrow windows while low potency compounds will have a larger selectivity window. Therefore, the clinical impact of JAK isoform selectivity is dependent on dose, cell type, tissue penetration and the genetics of the individual patient. This is probably best illustrated by the change in Hb in tofacitinib 5 mg-treated patients, with less Hb increase in the 10 mg group.

**Table 4:** *In vitro* JAK isoform selectivity

	Enzyme essay IC50 (nM)						
Compound	JAK1	JAK2	JAK3	TYK2	JAK2:JAK1	JAK3:JAK1	TYK2:JAK1
Tofacitinib	15.1	77.4	55.0	489	5.1	3.6	32.4
Baricitinib	4.0	6.6	787	61	1.5	196.8	15.3
Filgotinib	363	2400	>10 000	2600	6.6	>27.5	7.2
Upadacitinib	8	600	139	NA	75	17.4	NA
Peficitinib	3.9	5.0	0.7	4.8	1.3	0.2	1.2
Decernotinib	112	619	74.4	>10 000	5.5	0.67	>89

JAK: Janus kinase; IC50: half maximal inhibitory concentration; TYK2: Non-receptor Tyrosine-protein Kinase 2.

#### JAK isoforms as therapeutic targets in RA

Since JAK isoforms are important in the signal transduction of multiple cytokines, different JAKi might inhibit different cytokines, which could be important in tailoring treatment to patients. All current JAKi approved and in development have a significant effect on JAK1. Even for the JAK3-selective decernotinib, the ratio between JAK3 and JAK1 inhibition is only 0.6. JAK1 is involved in the signalling transduction of IL-6, IFN and the common γ -chain cytokines including IL-2 and IL-15. Among these, IL-6 is a proven therapeutic target in RA. Some of the side effects associated with IL-6 inhibition, such as increase in liver transaminases and lipids, neutropaenia as well as gastrointestinal perforation, are also associated with JAKi, suggesting that inhibiting IL-6 is a significant mode of action. However, JAKi differ from IL-6 inhibitors in that CRP, while reduced, did not normalize, suggesting that inhibition of IL-6 activity is incomplete [39]. A phase II RCT with very small patient numbers suggested that anti-IFN-γ may be effective in RA [56]. Whether IFN suppression is an important contributor to the therapeutic benefit of JAK1 inhibitors is unclear. However, IFN-γ is important in anti-viral immunity, which is a potential explanation for herpes zoster reactivation. Nevertheless, current evidence suggests that JAK1 is an important therapeutic target in RA.

Whether JAK2 is a good therapeutic target in RA is less clear. Pro-inflammatory cytokines such as GM-CSF signal via JAK2. A phase II RCT of anti-GM-CSF receptor α mAb showed benefit in RA [57]. A head-to-head trial suggested of anti-GM-CSFα mAb was as efficacious as TNF inhibitor [58]. Therefore, inhibiting both JAK1 and JAK2 may produce a greater anti-inflammatory effect by inhibiting GM-CSF. The disadvantage of inhibiting JAK2 is potential side effects associated with inhibiting haematopoetic cytokines including erythropoietin, IL-3 and IL-5, and hormones such as prolactin and growth hormone. The clinical significance of long-term JAK2 inhibition remains unknown. It will be prudent to investigate whether JAK2 inhibition leads to increase in platelet count and the rare cases of DVT/PE. *In vitro* experimentation has shown that JAK2 is involved in platelet activation. JAK2 inhibitor attenuated collagen-induced platelet aggregation in a dose-dependent manner [59], so an association between thrombosis and JAK2 via effect on platelet seems counterintuitive. Aside from the hitherto unknown effect of JAK2 inhibition and thrombosis, small molecule inhibitors, unlike mAbs, are more likely to have off-target side effects that may not be associated with JAK inhibition but are compound specific.

Based on available data, it is unclear whether JAK3 and TYK2 are desirable therapeutic targets in RA.

## Conclusion

JAK isoform selectively of JAKi is relative and not absolute. It is dose and tissue dependent.

Current approved JAKi and those in development significantly inhibit JAK1, which is an effective therapeutic target in RA, although herpes zoster reactivation is probably a class effect of JAK1 inhibitors. The balance of benefit and risk of inhibiting JAK2, JAK3 and TYK2 is uncertain and should be evaluated in the future. The exact role of JAKi in the treatment pathway of RA should be further assessed by head-to-head and strategy trials. The manufacturing cost of JAKi is substantially less than biologics. With the potential of generic JAKi in the future, widening access to more effective treatment for RA is becoming tangible.
